# Factors Affecting the Exercise Capacity in Pediatric Primary Hypertension

**DOI:** 10.3389/fped.2022.882223

**Published:** 2022-05-25

**Authors:** Hui Zhang, Yeshi Chen, Tong Zheng, Mingming Zhang, Xiaohui Li, Lin Shi

**Affiliations:** ^1^Department of Cardiovascular Medicine, Children's Hospital Capital Institute of Pediatrics, Peking Union Medical College Graduate School, Beijing, China; ^2^Capital Institute of Pediatrics-Peking University Teaching Hospital, Beijing, China; ^3^Department of Cardiovascular Medicine, Children's Hospital Capital Institute of Pediatrics, Beijing, China

**Keywords:** primary hypertension, children, treadmill exercise test, exercise capacity, cardiology, pediatrics

## Abstract

**Purpose:**

Exercise training is crucial to the early intervention of pediatric primary hypertension (PHT). However, much less is known about exercise capacity in this disease. This work investigated the exercise capacity in pediatric PHT and analyzed the factors affecting exercise capacity.

**Methods:**

The study enrolled children with PHT at the Children's Hospital Capital Institute of Pediatrics between July 2017 and July 2020. The Bruce protocol of the treadmill exercise test (TET) was used to assess exercise capacity. Multivariate ordinal logistic regression and generalized linear models were used to analyze factors affecting exercise capacity.

**Results:**

Of 190 patients, 146 (76.8%) were male, and the median age was 13 (11, 14). Most children accomplished TET and achieved the submaximal heart rates (189 [99.5%]). Children with lower resting diastolic blood pressure (DBP) and 24 h average diastolic blood pressure (ADBP) could achieve a TET stage of 6 or more, whereas children with higher DBP and ADBP could only achieve a TET stage of 3 (*P* all < 0.05). Children with lower DBP and 24 h ADBP were also associated with greater metabolic equivalents (METs; *r* = −0.237, *r* = −0.179, *P* all < 0.05). The completion of TET stages was negatively associated with female (OR = 0.163), younger age (OR = 1.198), greater body mass index (BMI, OR = 0.921), and higher 24 h ADBP (OR = 0.952, *P* all < 0.05). In addition, METs were negatively associated with female (β = −1.909), younger age (β = 0.282), greater BMI (β = −0.134), and higher 24 h ADBP (β = −0.063, *P* all < 0.05).

**Conclusions:**

Exercise capacity was impaired among pediatric PHT patients. Female gender, younger age, greater BMI, and higher 24 h ADBP are independently associated with the exercise capacity in pediatric PHT. These findings may help developing scientific exercise prescriptions for pediatric PHT.

## 1. Introduction

Hypertension, as a major risk factor for cardiac events and kidney diseases, is surprisingly common in pediatric population. A recent study established that the overall prevalence of hypertension in children and adolescents aged 0–18 years is 3–5% (1). Historically, the most prevalent form of hypertension in childhood used to be secondary hypertension. This situation has changed within the last two decades. PHT is now the dominant cause of hypertension in children above 6, especially in adolescents (2). Although the exact prevalence of pediatric PHT remains unknown, some studies showed that PHT accounted for 43% of pediatric hypertension in the US and 21.2–78% of cases in China (2–4). Childhood hypertension is likely to pose long-term damage, increasing the prevalence and severity of hypertension in adulthood (5). Therefore, active control of childhood PHT is far-reaching. Exercise intervention is a primary treatment for pediatric PHT (6, 7). So far, no validated exercise prescription is available in children with PHT, so this is a high unmet need. However, the development of exercise prescriptions is hampered by limited knowledge of exercise capacity in pediatric PHT.

Treadmill exercise test (TET) is a non-invasive test frequently used to measure exercise capacity by regulating the exercise load of participants with the alteration of treadmill movements (8). The indicators reflecting cardiac function, such as blood pressure (BP), heart rate, exercise stage achieved, and metabolic equivalents (METs), are monitored throughout the test and could be related to patients' exercise capacity.In this study, we evaluated the exercise capacity in pediatric PHT by TET and identified the factors affecting the exercise capacity in this disease.

## 2. Methods

### 2.1. Patient Population

We conducted a retrospective study of children with PHT at the Children's Hospital Capital Institute of Pediatrics between July 2017 and July 2020. The diagnosis of pediatric PHT was based on the Current Clinical Practice Guideline on Pediatric hypertension in Children and Adolescents Pediatrics (9) and the Chinese Guidelines for the Prevention and Treatment of Hypertension (6). In brief, systolic blood pressure (SBP) and diastolic blood pressure (DBP) should be ≥ 95th percentile in children with the same age, sex, and height for 3 consecutive records, and 2 weeks' interval is required between each recording. Meanwhile, patients with secondary hypertension were excluded. Hypertension was categorized: for children <13 years of age, stage 1 hypertension was defined as ≥95th percentile to <95th percentile + 12 mmHg (on the basis of age, sex, and height percentiles), or 130/80 to 139/89 mmHg (whichever is lower), and stage 2 hypertension was defined as ≥ 95th percentile + 12 mmHg, or ≥ 140/90 mmHg (whichever is lower); for children ≥ 13 years of age, stage 1 hypertension was defined as 130/80 to 139/89 mmHg, and stage 2 hypertension was defined as ≥ 140/90 mmHg (9). Exclusion criteria included patients with severe hypertension (SBP > 200 mm Hg and/or DBP > 120 mm Hg); patients with a lower-extremity injury who could not perform TET; and patients who had used any medication before TET. This study was approved by the Ethics Committee of the Capital Institute of Pediatrics (No. SHERLL2019003). Informed consent was signed by the parents and guardians of all subjects.

### 2.2. Treadmill Exercise Test

Before testing, the subjects would rest for 15–20 min, during which bodyweight, height, 12-lead ECG, and resting BP were measured. Exercise capacity was then assessed by a graded exercise test on the treadmill (Guangzhou Dimao Information Technology Co. TM-18). The speed and slope of the treadmill sequentially increased to adjust the exercise load volume. The endpoint of the TET was reaching the target heart rate [submaximal target heart rate = (220 - age) × 85%]. To prevent accidents, the test would be terminated immediately if any of the indications for termination happened. Participants were continuously being monitored during the 8-min rest after TET.

Our method followed the Bruce protocol (8, 10) with two minor modifications: the time of each exercise stage was shortened to 1–2 min, and the overall duration of exercise was controlled at 8–11 min. Accordingly, exercise in TET was graded 1–7 (Table 1). Higher stages of exercise signify better exercise capacity.

**Table 1:** Revised Bruce protocol.

**Stages**	**Speed (km/h)**	**Slope (%)**	**Time (min)**
1	2.8	10	1–2
2	4.0	12	1–2
3	5.5	14	1–2
4	6.7	16	1–2
5	8.0	18	1–2
6	8.8	20	1–2
7	9.6	22	1–2

### 2.3. Assessment of Metabolic Equivalents

Metabolic equivalents were used to describe the exercise stress at the endpoint of TET. One MET is the amount of oxygen consumed by the body while sitting at rest, which is approximately 3.5 mL O^2^/kg/min (11). Larger METs values indicate higher intensity of exercise and better exercise capacity (12). The METs was calculated as following:


$$\begin{array}{l} METs=[(\frac{speed}{6.0}+speed\times slope\times \frac{3.0}{1000.0})÷3.5\\ \;+1-last\;second\;METs]\times \frac{j}{120.0}+last\;second\;METs \end{array}$$

*j*: total time per stage, time in seconds.

### 2.4. Assessment of the Cardiac Involvement

Echocardiograms were measured using a Phillips iE33 (Phillips, The Netherlands). Left ventricular end-diastolic diameter, interventricular septal end-diastolic thickness, and left ventricular posterior wall thickness were measured from the parasternal long-axis view. Left ventricular mass (LVM) was calculated according to the formula recommended by Devereux et al. (13). Left ventricular hypertrophy (LVH) was defined as LVMI ≥ 45g/m^2.7^ in boys, and LVMI ≥ 40 g/m^2.7^ in girls (14).

### 2.5. Statistical Methods

For the normal continuous variables, the *t*-tests were done to compare two groups; the one-way ANOVA were done to compare multiple groups; and the least significant difference tests were done to compare variables within one group. For the non-normal continuous variables, Wilcoxon rank-sum tests were done to compare two groups; Kruskal–Wallis *H*-tests were used to compare multiple groups; Spearman correlation coefficients analysis was done to analyze the connection between two groups; and partial correlation analysis was done to analyze the connection between two groups when corrected with cofounders. Variables were described as mean ± SD (or median [P_25_, P_75_] for non-normal data). If needed, baseline data were compared between different groups to assess for imbalance. Any data that was unbalanced at baseline and associated with the results were included in the partial correlation model and logistic regression model as cofounders.

To analyze factors associated with exercise capacity, the multivariate ordinal logistic regression was used to identify the factors affecting patients' completion of TET stages, and the generalized linear model was used to identify the factors affecting METs. The dependent variables of the models were TET stages and METs, respectively; age, sex, BMI, LVH, and ADBP were included as influencing factors. P-values <0.05 were considered statistically significant. Statistical analysis was performed using IBM SPSS 22.0.

## 3. Results

### 3.1. Characteristics of Patient Population

A total of 190 children with PHT were included in this study. There was a male dominance (76.8%), the median age was 13 years (11 years,14 years), and 189 patients (99.5%) achieved the target heart rate and completed the TET. Only 1 child (0.5%) terminated the TET prematurely at minute 4 due to dizziness and unsteadiness. The patient was a 14-year-old girl with stage 2 PHT and LVH and had no anomaly in her echocardiogram or resting ECG.

Of 189 patients completed the TET, 90 (47.6%) had stage 1 PHT (61 [67.8%] were boys), 99 (52.4%) had stage 2 PHT (85 [85.9%] were boys), 184(97.4%) did not have LVH (144 [78.3%] were boys), and 5(2.6%) had LVH (2 [40.0%] were boys).

### 3.2. Blood Pressure and Exercise Capacity

A comparison of the baseline data showed significant differences in gender and body mass index (BMI; *p* < 0.05) between children with stage 1 and stage 2 hypertension.

An analysis of correlation between hypertension stages and exercise capacity was done. The result showed that the TET stage patients achieved (*p* = 0.250) and the METs (*p* = 0.109) did not differ between children with stage 1 and stage 2 hypertension when adjusted for age, gender and BMI (Table 2), suggesting that there was no significant difference in exercise capacity between children with various hypertension stages.

**Table 2:** Comparison of exercise capacity between different hypertension stages.

		**Stage 1 PHT (*n* = 90)**	**Stage 2 PHT (*n* = 99)**	***P***	***P'***
**Clinical characteristics**					
Age (years)		13 (11,14)	13 (11,14)	0.17	-
Sex (male [%])		61 (67.8%)	85 (85.9%)	0.003	-
BMI		25.6 ± 4.5	28.1 ± 4.6	<0.001	-
Mean ± SD(kg/m^2^)					
**Exercise capacity**					
METs		10.2 (8.3, 11.3)	9.3 (8.2, 11.3)	0.317	0.109
TET stages	3 n (%)	6 (37.5%)	10 (62.5%)	0.419	0.25
	4 n (%)	41 (50.6%)	40 (49.4%)		
	5 n (%)	32 (43.2%)	42 (56.8%)		
	≥ 6 n (%)	11 (61.1%)	7 (38.9%)		

*P', P-value after corrected with age, gender, and BMI; PHT, primary hypertension; BMI, body mass index; METs, metabolic equivalents; TET, treadmill exercise test*.

*Data are presented as median[P_25_,P_75_], except where otherwise indicated*.

**Figure 1:**
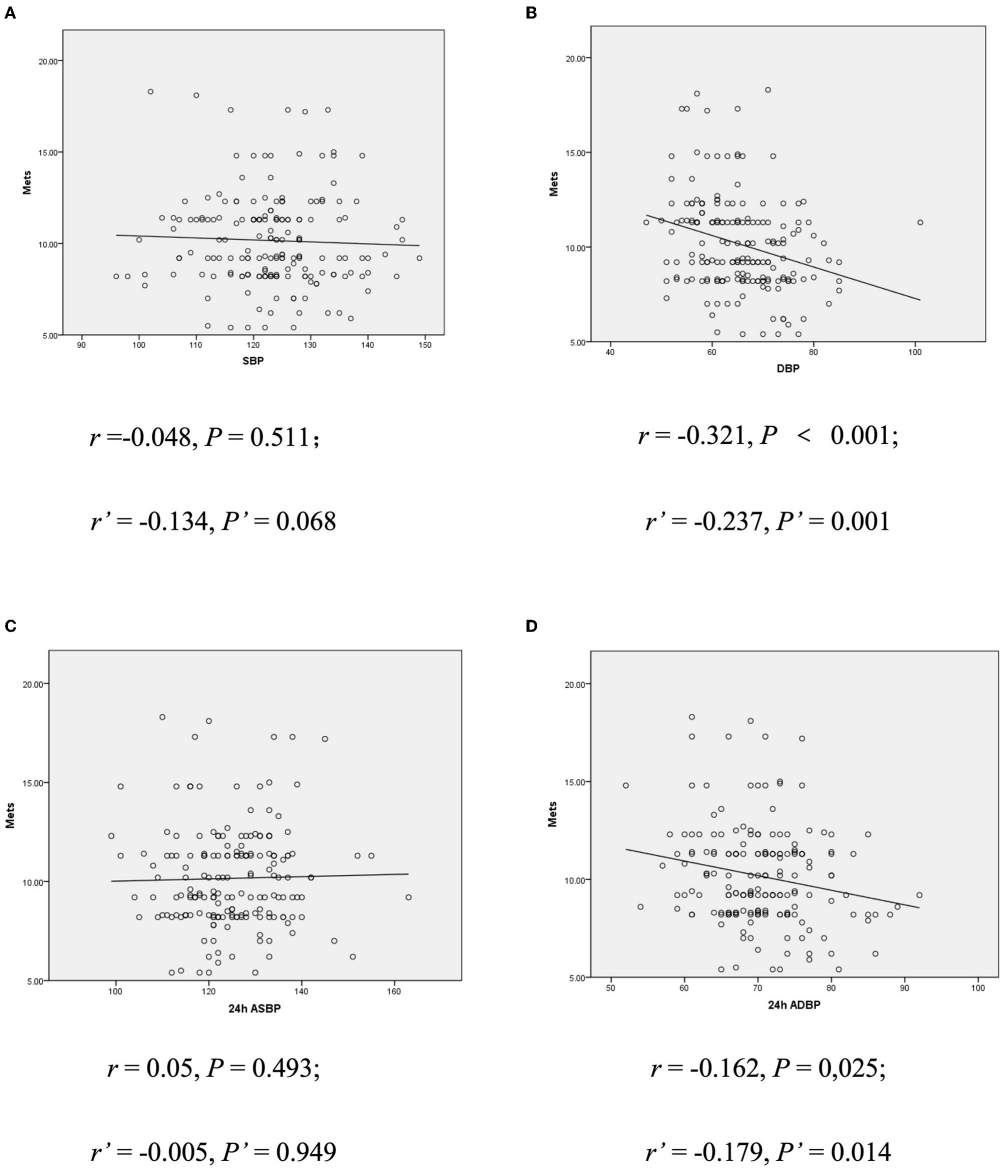
**(A)** Correlation between resting SBP and METs. **(B)** Correlation between resting DBP and METs. **(C)** Correlation between 24 h ASBP and METs. **(D)** Correlation between 24 h ADBP and METs. r, Spearman rank correlation coefficient; r', bias correlation coefficient corrected with gender and BMI; P', *P*-value when corrected with age, gender, and BMI; DBP, diastolic blood pressure; METs, metabolic equivalents; ASBP, average systolic blood pressure.

Further analysis of correlation between BP value and exercise capacity showed that children with lower resting DBP and 24 h average blood pressure (ADBP) could achieve a TET stage of 6 or greater, but those with higher DBP and 24 h ADBP could only achieve a TET stage of 3 (*p* < 0.05). The results were consistent when corrected for age, gender and BMI (*p* < 0.05, seen in Table 3). In addition, METs was negatively correlated with resting DBP (*r* = −0.321; *p* < 0.001) and 24 h ADBP (*r* = −0.162; *p* < 0.05). After adjustment for age, gender and BMI, the correlation between METs and resting DBP (*r*' = −0.237; *p* < 0.05) and 24 h ADBP (*r*' = −0.179; *p* < 0.05) hardly changed (Figure 1). Meanwhile, there was no significant difference in SBP between children with different exercise capacity.

**Table 3:** Comparison of blood pressure value between different TET stages.

		**Resting SBP (mmHg)**	**Resting DBP (mmHg)**	**ASBP (mmHg)**	**ADBP (mmHg)**
TET stages	3 (*n* = 16)	124 (120, 129)	70 (62, 74)	124 (119, 133)	74 (69, 80)
	4 (*n* = 81)	123 (118, 129)	67 (61, 72)	122 (117, 131)	70 (67, 74)
	5 (*n* = 74)	123 (117, 127)	63 (57, 68)	127 (119, 133)	71 (66, 75)
	≥6 (*n* = 18)	125 (118, 133)	60 (56, 67)^#^	128 (116, 133)	70 (63, 72)*
P		0.810	0.003	0.342	0.034
P'		0.121	0.012	0.905	0.026

*P', *P*-value after corrected with age, gender, and BMI; SBP, systolic blood pressure; DBP, diastolic blood pressure; ASBP, average systolic blood pressure; ADBP, average diastolic blood pressure*.

*#The lower resting DBP of patients achieved stage ≥6 TET compared with that of patients achieved stage 3TET*.

*The lower ADBP of patients achieved stage ≥6 TET compared with that of patients achieved stage 3 TET.

*Data are presented as median[P_25_,P_75_], except where otherwise indicated*.

### 3.3. LVH and Exercise Capacity

Analyses were performed to explore the difference in exercise capacity between the children with (*n* = 5) and without LVH (*n* = 184). There was no significant difference in the METs nor TET stages (*p* > 0.05). Factors associated with exercise capacity in this study (including age, sex, and BMI) were adjusted in the comparison, after which no significant differences in the TET stages (*p* = 0.610) nor METs (*p* = 0.609) were detected (Table 4).

**Table 4:** Comparison of exercise capacity between patients with and without left ventricular hypertrophy.

		**Without LVH (*n* = 184)**	**With LVH (*n* = 5)**	***P***	***P'***
**Clinical characteristics**					
Age (years)		13 (11, 14)	14 (13, 14)	0.248	-
Sex (male; %)		144 (78.3%)	2 (40%)	0.079	-
BMI (kg/m^2^)		26.5 (23.0, 29.7)	34.5 (29.3, 39.3)	0.008	-
**Exercise capacity**					
METs		9.6 (8.3, 11.4)	9.2 (6.9, 11.3)	0.474	0.609
TET	3 n (%)	15 (93.8%)	1 (6.2%)	0.456	0.610
	4 n (%)	79 (97.5%)	2 (2.5%)		
	5 n (%)	72 (97.3%)	2 (2.7%)		
Stages	≥6 n (%)	18 (100%)	0 (0%)		

*P', *P*-value corrected with age, gender, and BMI; BMI, body mass index; LVH, left ventricular hypertrophy; TET, treadmill exercise test; METs, metabolic equivalents*.

*Data are presented as median[P_25_,P_75_], except where otherwise indicated*.

### 3.4. Factors Affecting Exercise Capacity

In the multivariate ordinal logistic regression, there was collinearity between resting DBP and 24 h ADBP, thus 24 h ADBP was included in the model. The results showed that the completion of TET stages was positively associated with age (OR = 1.198, 95% CI 1.052 to 1.364, *p* < 0.05) and negatively associated with female gender (OR = 0.163, 95% CI 0.079 to 0.333, *p* < 0.05), BMI (OR = 0.921, 95% CI 0.863 to 0.981, *p* < 0.05), and 24 h ADBP (OR = 0.952, 95% CI 0.912 to 0.993, *p* < 0.05; Table 5).

**Table 5:** Multivariate analysis regarding TET stages in children with PHT.

**Indicators**	**TET stages**				Multivariate*		
	**3 (*n* = 16)**	**4 (*n* = 81)**	**5 (*n* = 74)**	**≥6 (*n* = 18)**	**OR**	**95%CI (OR)**	***P-value***
Age (years)	13 (12, 14)	12 (11, 14)	13 (11, 14)	13 (12, 14)	1.198	1.052, 1.364	0.007
Sex
Girls (%)	7 (43.7)	28 (34.6)	8 (10.8)	0 (0.0)	0.163	0.079, 0.333	<0.001
Boys (%)	9 (56.3)	53 (65.4)	66 (89.2)	18 (100.0)	Ref	Ref	
BMI (kg/m^2^)	29.93 (25.90, 32.89)	26.56 (23.08, 30.27)	26.51 (23.08, 30.27)	25.67 (23.08, 30.27)	0.921	0.863, 0.981	0.01
Without LVH (%)	15 (93.8%)	79 (97.5%)	72 (97.3%)	18 (100%)	0.586	0.095, 3.613	0.563
With LVH (%)	1 (6.2%)	2 (2.5%)	2 (2.7%)	0 (0%)	Ref	Ref	
ADBP (mmHg)	74 (69,80)	70 (67,74)	71 (66,75)	70 (63,72)	0.952	0.912, 0.993	0.025

*OR, odds ratio; 95%CI, 95% confidence interval; Ref, reference value; BMI, body mass index; LVH, left ventricular hypertrophy; ADBP, average diastolic blood pressure*.

*Data are presented as median[P_25_,P_75_], except where otherwise indicated*.

*The multivariate ordinal logistic regression analysis model included TET stages as dependent variable and age, sex, BMI, LVH, and ADBP as independent variables.

The analyses of generalized linear model showed that METs, similar to TET stages, were positively associated with age (β = 0.282, 95%CI 0.134 to 0.429, *p* < 0.05) and negatively associated with female gender (β = −1.909, 95% CI −2.672 to −1.146, *p* < 0.05), BMI (β = −0.134, 95% CI −0.205 to −0.063, *p* < 0.05), and 24 h ADBP (β: −0.063, 95%CI: [−0.112, −0.014], *p* < 0.05; Table 6).

**Table 6:** Multivariate analysis regarding METs in children with PHT.

**Indicators**	**Univariate**			Multivariate*		
	**β**	**95%CI**	***P-value***	**β**	**95%CI**	***P-value***
Age	0.168	0.006,0.329	0.041	0.282	0.134, 0.429	<0.001
Sex
Girls	−1.755	−2.576, −0.943	<0.001	−1.909	−2.672, −1.146	<0.001
Boys	Ref	Ref		Ref	Ref	
BMI(kg/m^2^)	−0.13	−0.203, −0.057	<0.001	−0.134	−0.205, −0.063	<0.001
Without LVH	1.089	−1.126, 3.304	0.335	−0.663	−2.714, 1.388	0.526
With LVH	Ref	Ref		Ref	Ref	
ADBP (mmHg)	−0.075	−0.127, −0.022	0.005	−0.063	−0.112, −0.014	0.011

*β, regression coefficient; 95%CI, 95% confidence interval; Ref, reference value; BMI, body mass index; LVH, left ventricular hypertrophy; ADBP, average diastolic blood pressure*.

*The generalized linear model analysis model included METs as dependent variable and age, sex, BMI, LVH, and ADBP as independent variables.

## 4. Discussion

This study evaluated exercise capacity in pediatric PHT through TETs. In the study, 99.5% of children with PHT completed TETs and achieved the submaximal heart rate, indicating that exercise interventions could be applied to most pediatric PHT patients. Among children who completed TETs, exercise capacity was negatively correlated with DBP and 24 h ADBP. Furthermore, lower exercise capacity was associated with female gender, younger age, greater BMI, and higher 24 h ADBP.

Exercise training was recommended as a primary non-pharmacological intervention which should be implemented in all hypertensive patients (4). A meta-analysis reported that aerobic exercise training could effectively reduce mean SBP and DBP (15). However, the “optimal” dose of exercise training for BP control, which is critical to maximize intervention efficiency (16), has not been defined. Hansen et al. (17) suggested that higher exercise intensity was associated with greater effectiveness of aerobic exercise training, while another research showed that moderate-intensity resistance training may lead to similar BP reductions (18). Moreover, according to Aslani et al. (19), very strenuous prolonged exercise could raise the possibility of cardiac fatigue. To find the most suitable exercise intensity for children with PHT, we raised an investigation on the exercise capacity of these children.

In our study, we noticed that children with higher BP had worse exercise capacity. At one level, children with lower DBP and 24 h ADBP could achieve higher TET stages. On another scale, METs were also negatively correlated with DBP and 24 h ADBP levels. Note that SBP did not differ between different groups, suggesting that DBP might be a better predictor of poor exercise capacity than SBP, especially in young populations. Interestingly, DBP has also been regarded as the strongest predictor of coronary heart disease in younger populations (<50 years) (20). A possible explanation for this might be that young patients are less likely to suffer from the peripheral amplification of SBP caused by wave reflection, which is the main reason for the age-related BP changes in the hypertensive population (21). As both poor exercise capacity and coronary heart disease lead to negative outcomes, DBP is thereby a potential predictor for worse prognoses of pediatric PHT.

Despite the inconsistency in DBP and SBP, our finding of the link between BP and exercise capacity are in accord with the studies in adults (22, 23), which showed that the exercise capacity of the hypertensive group was significantly lower than that of the healthy control group. The studies also suggested that the deterioration of exercise capacity in hypertensive patients began long before the alteration of cardiac structure. The lower exercise capacity may prevent patients from high-intensity training. This relatively poor exercise capacity also intrigued us to the factors affecting exercise capacity in pediatric PHT, which were then explored by multifactor analyses.

An unexpected result of the analyses was the lack of association between LVH and METs. This result is contradictory to previous studies, which showed that exercise capacity was negatively related to LVH in hypertensive patients (22, 24). The relationship may be explained by the shortage of blood flow in coronary and non-coronary arteries and myocardial blood reserve in patients with hypertension with LVH. We presume that the inconsistency of the results may be attributed to the relatively small sample size of patients with LVH.

Furthermore, we found that girls had significantly lower exercise capacity than boys, which was consistent with healthy children (25). The results may be explained by the relatively lower oxygen uptake of females, which may impair their exercise capacity (26). This lower oxygen uptake is brought by their smaller muscle mass, hemoglobin and blood volume, and stroke volume (8).

Our data also suggest a positive association between exercise capacity and age, a finding similarly reported by Cumming et al. (25) with healthy children and Ulrich et al. (27) when using the 6-min walk test (6MWT) to estimate the exercise capacity in healthy children and adolescents. The result is contradicted to the findings in adults (8). This discrepancy may be due to that the pediatric population is in a stage of growth and development, and hence their physical strength and exercise capacity develop with age.

It is generally believed that children's BMI is related to their exercise capacity. Previous studies show that obese children falter in several aspects of exercise capacity, including muscle explosive strength, muscle endurance, and running speed (28, 29). Obesity also poisons hemodynamic parameters, according to a study by Fornitano et al. (30). Our study found that BMI was related to exercise capacity in children with PHT. The METs of the patients and the TET stage they could achieve dropped with the increase of BMI. One possible reason for this anticorrelation is that the overweight populations have a hypoadrenergic state during exercise (31). Moreover, obesity could impair exercise-related physiological functions, such as lung function (32, 33), oxygen saturation (34), cardiovascular function (35), and the biological function of skeletal muscle(36).

Twenty-four hours ADBP was negatively associated with exercise capacity in our study, indicating that exercise capacity declines with the increase of BP. A similar result was shown in adults: 43.9% of hypertensive patients failed to achieve the predictive distance of 6WMT (37). An animal model also suggested that hypertensive subjects had lower exercise capacity than healthy ones (38). A possible explanation for this might be that higher BP results in higher peripheral resistance, causing a greater cardiac output during exercise, resulting in the decline in exercise capacity.

Our study had several limitations. One was the small sample size in single center, especially of the LVH group, which is due to the rather mild condition of our patients. The study was also limited by the lack of a healthy control group, given the difficulty in recruiting volunteers during the COVID pandemic. An additional problem is that pulmonary function was not evaluated in our study, which should be included in future studies to provide a comprehensive assessment of exercise capacity.

## 5. Conclusions

Our study showed that exercise capacity was impaired in pediatric PHT, due to the higher DBP and 24 h ADBP. We also found that female gender, younger age, greater BMI, and higher 24 h ADBP were both independently associated with lower exercise capacity in pediatric PHT. Future prospective studies with a larger sample size and healthy control may be of use.

## Data Availability Statement

The original contributions presented in the study are included in the article/supplementary material, further inquiries can be directed to the corresponding author/s.

## Ethics Statement

The studies involving human participants were reviewed and approved by the Ethics Committee of the Capital Institute of Pediatrics. Written informed consent to participate in this study was provided by the participants' legal guardian/next of kin.

## Author Contributions

HZ collected the data, performed the data analyses, and drafted the paper. YC wrote the manuscript. TZ carried out the treadmill exercise test and performed the analyses. MZ contributed to data collection. XL designed the study and revised the manuscript. LS monitored data collection for the trial. All authors contributed to the article and approved the submitted version.

## Funding

XL was supported by Beijing Hospital Administration's Peak Climbing Talents Program (DFL20181301). LS was supported by the Beijing Municipal Hospital Administration, Pediatric Special General Project of the Collaborative Development Center of Pediatrics (XTYB201801).

## Conflict of Interest

The authors declare that the research was conducted in the absence of any commercial or financial relationships that could be construed as a potential conflict of interest.

## Publisher's Note

All claims expressed in this article are solely those of the authors and do not necessarily represent those of their affiliated organizations, or those of the publisher, the editors and the reviewers. Any product that may be evaluated in this article, or claim that may be made by its manufacturer, is not guaranteed or endorsed by the publisher.
